# Items

**Published:** 1881-09

**Authors:** 


### Items.

OFFICIAL LIST OF MEDICAL OFFICERS AND ACTING ASSISTANT SURGEONS of the United States Marine Hospital Service, with their Stations, July 1, 1881:

*Supervising Surgeon-General.*—John B. Hamilton, Washington, D. C.

*Surgeons.*—Ernest Hebersmith, San Francisco, Cal.; P. H. Bailhache, National Board of Health; John Vansant, Boston, Mass.; W. H. H. Hutton, Detroit, Mich.; T. W. Miller, Chicago, Ill.; Walter Wyman,* Cincinnati, Ohio; W. H. Long, Louisville, Ky.; R. D. Murray, Memphis, Tenn.; C. S. D. Fessenden, New York, N. Y.; George Purviance, Pittsburgh, Pa.; H. W. Sawtelle St. Louis, Mo.; H. W. Austin, New Orleans, La.; J. M. Gassaway, Philadelphia, Pa.

*Passed Assistant Surgeons.*—Henry Smith, Norfolk, Va.; G. W. Stoner, Portland, Me.; J. C. Fisher, Washington, D. C.; John Godfrey, Mobile, Ala.; C. B. Goldsborough, Baltimore, Md.

*Assistant Surgeons.*—Fairfax Irwin, Wilmington, N. C.; F. W. Mead, Port Townsend, W. T.; H. P. Cooke, Galveston, Texas; H. R. Carter, Cairo, Ill.; W. H. Heath, Buffalo, N. Y.; F. J. O'Connor, Evansville, Ind.; F. D. Porter, Chicago, Ill.; John Guiteras, Key West, Fla.; W. A. Wheeler, Charleston, S. C.; J. A. Benson, St. Louis, Mo.; C. E. Banks, San Francisco, Cal.; D. A. Carmichael, New York, N. Y.; S. T. Armstrong, New Orleans, La.

*Acting Assistant Surgeons.*—J. M. Allen, Milwaukee, Wis.; W. B. Banks, Rockland, Maine; H. G. Bates, New Berne, N. C.; C. C. Berry, Portland, Maine; R. D. Bibber, Bath, Maine; F. N. Blount, Pensacola, Fla.; J. E. Bready, Dubuque, Iowa; G. B. Case, Cleveland, Ohio; Byron DeWitt, Oswego, N. Y.; L. B. Edwards, Richmond, Va.; A. W. Fisher, Toledo, Ohio; J. P. C. Foster, New Haven, Conn.; W. M. Griffiths,* Louisville, Ky.; A. C. Hamlin, Bangor, Maine; L. W. Hodgkins, Ellsworth, Maine; S. B. Hunter, Machias, Maine; J. M. Kercheval, Nashville, Tenn.; H. E. Mereness, Albany, N. Y.; J. D. Mitchell, Jacksonville, Fla.; Charles Ottilie, La Crosse, Wis.; R. H. Pease, Chicago, Ill.; C. T. Peckham,† Boston, Mass.; W. H. Pope, Fernandina, Fla.; T. T. Price, Tuckerton, N. J.; S. D. Robbins, Vicksburgh, Miss.; Elmer Small, Belfast, Maine; J. G. Stanton, New London, Conn.; W. D. Stewart, Vineyard Haven, Mass.; G. H. Stone, Savannah, Ga.; D. H. Strickland, Erie, Pa.; H. S. Taft, Marquette, Mich.; W. H. Taylor, New Bedford Mass.; J. H. Vandeman, Chattanooga, Tenn.; M. F. Wentworth, Portsmouth, N. H.; C. A. Wheaton, Jr., St. Paul, Minn.; J. E. Wood, Elizabeth City, N. C.

* Temporary duty as medical officer revenue-bark "Chase."

† Temporary duty at Cincinnati during the absence of Surgeon Wyman.

‡ Temporary.

OFFICIAL LIST OF CHANGES OF STATIONS AND DUTIES OF MEDICAL OFFICERS of the U. S. Marine Hospital Service, April 1, 1881, to June 30, 1881:

*Bailhache, P. H.*, Surgeon. Detailed as Chairman Board of Examiners. April 5, 1881.

*Wyman, Walter*, Surgeon. Detailed for temporary duty as medical officer revenue-bark "Chase." May 31, 1881.

*Long, W. H.*, Surgeon. Detailed as member Board of Examiners. April 5, 1881.

*Murray, R. D.*, Surgeon. To proceed to Memphis, Tenn., assume charge of the service at that port, and inspect the service at Vicksburg, Miss. April 8, 1881.

*Purviance, George*, Surgeon. Detailed as Recorder Board of Examiners. April 5, 1881. To proceed to Richmond, Va., as inspector. April 30, 1881. Detailed as Chairman Board of Survey, to examine applicants for admission to the Revenue Marine Service. May 10, 1881.

*Doernig, E. J.*, Surgeon. Granted leave of absence for thirty days from April 21, 1881. April 2, 1881.

*Austin, H. W.*, Surgeon. Granted leave of absence for thirty days from May 5, 1881. April 2, 1881.

*Gassaway, J. M.*, Passed Assistant Surgeon. To proceed to Philadelphia, Pa., and assume charge of the service, relieving Passed Assistant Surgeon Stoner. April 7, 1881.

*Smith, Henry*, Passed Assistant Surgeon. To report to Chairman Board of Examiners. April 25, 1881.

*Stoner, G. W.*, Passed Assistant Surgeon. To proceed to Portland, Maine, and assume charge of the service, relieving Surgeon Doering. April 7, 1881.

*Fisher, J. C.*, Passed Assistant Surgeon. Detailed as Recorder Board of Survey, to examine applicants for admission to the Revenue Marine Service. May 10, 1881.

*Wheeler, W. A.*, Assistant Surgeon. Granted leave of absence for thirty days (he providing a substitute,) from July 7, 1881. June 23, 1881.

*Carmichael, D. A.*, Assistant Surgeon. To proceed to New York, N. Y., and report to Surgeon Fessenden for duty. April 7, 1781.

RESIGNATION.

*Doering, E. J.*, Surgeon. Resignation accepted by the Secretary, to take effect May 20, 1881. April 2, 1881.

PROMOTION.

*Gassaway, J. M.*, Surgeon. Promoted to be Surgeon from May 21, 1881. May 16, 1881.

---

### EXAMINATION PAPERS OF THE ILLINOIS STATE BOARD OF HEALTH.

CHICAGO, JUNE 29, 1881.

*Obstetrics—By A. L. Clark, M.D.*

1. Give the mechanism of the Occipito-Sacral position.

2. Define placenta previa, and give—*a.* Its diagnosis. *b.* Prognosis to mother and child. *c.* Treatment.

3. How will you Diagnose Pregnancy from Ovarian Tumor?

4. Give the contra-indications for the use of Ergot.

5. How can you diagnose Fibroid Uterine Tumor from Flexion of the Uterus?

6. What blood-vessels are contained in the Umbilical Cord?

7. What are the sympathetic signs of Pregnancy?

8. Give Pathology, Diagnosis, Prognosis and Treatment for "hour glass contractions" of the Uterus.

9. What stage of labor is attended with the greatest danger, and what is the danger?

10. Give the dangers in—*a.* The first stage of labor. *b.* The second stage of labor. *c.* The third stage of labor.

*Chemistry—By A. L. Clark, M.D.*

1. What is the number of Simple Chemical Elements?

2. What is Equivalent Weight?

3. Give the Chemical Symbol for Antimony, and give an Antimonial Preparation sometimes used in medicine.

4. Explain the combustion and flame of a candle.

5. Under what circumstances will a galvanic current be formed?

6. What is the specific heat of any body?

7. What takes place when any soluble Salt of Iron is prescribed or mixed with most vegetable Infusions or Tinctures?

8. Give the Chemical Symbol for the Monoxide of Nitrogen, and suggest the uses of the compound.

9. What is Specific Gravity?

10. Name ten Elementary Substances.

*Gynæcology—By R. Ludlam, M.D.*

1. What is the most frequent of all the Uterine displacements?

2. Give the diagnosis of Hysteria from Epilepsy.

3. How would you know a case of Ulceration of the Uterine Cervix from one of Cervical Endometritis?

4. What are the symptoms of Laceration of the Cervix?

5. What is the prognosis in Pelvic Cellulitis?

6. When, if ever, do Fibroid Tumors become malignant?

7. Is Leucorrhœa a symptom or a disease?

8. Give the symptoms and treatment of Prolapse of the Ovary.

9. What are the indications for the use of the Sponge Tent?

10. Name the diseases in which Menorrhagia is an almost invariable symptom.

*General Pathology—By R. Ludlam, M.D.*

1. What is Anæmia?

2. Give the general pathology of Fever.

3. What is the difference between Hypertrophy and Atrophy?

4. What do physicians understand by compensating Hypertrophy? Give an illustration.

5. Define the word diathesis, and name the most important Diatheses.

6. Describe the different stages of Cerebral Apoplexy.

7. Is Dropsy a symptom or disease, and why?

8. What is the pathology of Hemi-anæsthesia?

9. In the hepatization of Pneumonia, if chloride of sodium is deficient in the urine, where do we find it?

10. In intermittent Fever when would you apply the thermometer in order to get the highest range of temperature?

*Hygiene—By J. H. Rauch, M.D.*

1. What kinds and what amount of food should be furnished those sick with acute diseases? When and how should it be given?

2. What are disinfectants? Name the best. How do they act as such? How and when should they be used?

3. How would you prevent or limit the spread of Typhoid Fever, Diphtheria, Scarlatina?

4. In a case of Typhoid Fever, where would you look for its probable source or origin?

5. What effect would the drainage of the level prairies of the State have upon health?

6. How would you ventilate the school room? How much air space should each pupil have?

7. How would you resuscitate from the asphyxia of Drowning, Coal Gas, Chloroform or Ether?

8. What is Trichinosis? How can it be prevented?

9. Which do you prefer, humanized or non-humanized vaccine virus, and why?

10. How would you diagnose Small Pox from Measles, Scarlet Fever, Chicken Pox?

11. How would you prevent the spread of Small Pox?

12. How would you render privies and water closets innocuous?

13. How do you account for the increased mortality of the past six months?

14. What effect, upon the public health, will result from the recent overflows in this State?

15. Why should the registration of Births, Deaths and Marriages be enforced?

*Medical Jurisprudence—By J. H. Rauch, M.D.*

1. In a case involving legal questions, how would you conduct a post-mortem examination?

2. How would you conduct the examination of an insane person for commitment to an insane asylum?

3. State under what circumstances you would deem it necessary to hold a coroner's inquest.

4. How would you differentiate between Drunkenness and Apoplexy?

5. To what extent are physicians liable for the bad results of treatment of a fracture?

*Practice of Medicine—By John McLean, M.D.*

1. Is Pneumonia a specific fever, or a local inflammation with fever as a result? Give reasons why.

2. What are the varieties of Pneumonia?
3. How would you treat a typical case of Catarrhal Pneumonia?
4. How would you diagnose Bronchitis from Pneumonia?
5. How would you treat Bronchitis?
6. How would you diagnose Pleuritis from Bronchitis? From Pneumonia?
7. How would you treat Acute Pleuritis?
8. What would be your treatment if effusion followed Pleuritis?
9. What is Diphtheria? How would you treat a case?
10. How would you diagnose Measles from Scarlet Fever?
11. How would you treat Measles?
12. How would you treat Scarlet Fever?
13. What are the symptoms of Acute Gastritis?
14. How would you treat Dysentery?
15. How would you diagnose a case of Morbus Brightii?
17. What pathological condition is found in Cerebro Spinal Meningitis?
18. How would you treat a case?
19. What is the supposed cause of Malarial Fever?
20. How would you treat a Remittent Fever?

*Materia Medica and Therapeutics—By J. H. Rauch, M.D.*

1. What are—[1] Infusions, [2] Tinctures, [3] Fluid Extracts, [4] Waters, [5] Solutions?
2. What are—[1] Ointments, [2] Cerates, [3] Suppositories, [4] Spirits, [5] Oleo-Resins?
3. Give full Latin officinal name, opium strength, and doses of—[1] Black Drop, [2] Dover's Powder, [3] Laudanum, [4] Extract of Opium. [5] How does the Deodorized Tincture of Opium differ from Laudanum?
4. [1] Name and give the origin of the three officinal kinds of Aloes. [2] Which sort is best for medical use, and why? Name and give doses of three Aloëtic preparations. [4] Give the composition of Compound Cathartic Pills.

Sources, active principles, three preparations, and doses of each of the following drugs:

5. Belladonna
6. Capsicum.
7. Cinchona.
8. Senna.
9. Cinnamon.
10. Dose of—[1] Acid Hydrocyanicum Dilutum, [2] Camphora, [3] Chloral, [4] Elaterium, [5] Ext. Ergotæ Fluid, [6] Ext. Hyoscyami, [7] Iodoformum, [8] Oleum Ricini, [9] Tinctura Aconiti Radicis, [10] Tinct. Veratri Viridis.
11. Various methods of treating acute rheumatism. Expectant, Alkaline, Salicylic, Theories and result.
12. Giving details of carrying out the above.
13. Hypnostics—Opium, Chloral, Bromides, Lactucarium—Peculiarities of each.

14. Cathartics—Mention five different kinds. State peculiarities of action, and occasions on which you would wish to use each.

15. Name the Cinchona Alkaloids. What are their comparative values?

*Physiology.*

1. What is the specific gravity of normal urine?
2. How is gastric juice produced?
3. How is the peristaltic motion of the bowels produced?
4. How many sounds has the heart? And how do you distinguish them?
5. Is bile a recrementitious or excrementitious product, or both?
6. What are the functions of the pneumogastric nerve?
7. Describe the difference between a serous and mucous membrane.
8. What organ of the body commences growing about the 45th year?
9. What function does the pancreatic juice perform?
10. Why is venous blood darker than arterial blood?

*Anatomy.*

1. Name the principal fluids of the body.
2. Describe the circulation of the blood in the adult.
3. State the number of bones composing the human skeleton, and name those of the cranium, face and vertebral column.
4. State number and names of the several muscles of the eye?
5. What are the principal divisions of the human brain?
6. Name and describe the membranes of the brain and spinal cord.
7. State number and names of the cranial nerves.
8. Describe the sympathetic nerve.
9. What structures are cut and what artery is endangered in the operation for strangulated hernia?
10. Describe the regions of the abdomen, and state their contents.

*Surgery.*

1. Define malignant and benign tumors, and give examples of each.
2. How diagnose dislocation of humerus from fracture?
3. How reduce subglenoid dislocation of humerus?
4. State how you would diagnose, dress and treat a simple fracture of the leg.
5. When is a wound said to heal by first intention, and what conditions are necessary to such a result?
6. What are the indications for tracheotomy, and the dangers attending the operation?
7. What would you do in fracture of the skull without signs of depression?
8. What conditions and indications would demand the amputation of an injured limb?
9. Give general directions for local and constitutional treatment after amputations?
10. How would you administer the anæsthetics, ether and chloroform, and what do in case of suspended respiration during their administration?

